# Supplementary material for: Genetic studies with a uracil DNA glycosylase biosensor support a role for mitochondrial UNG1 in nuclear uracil repair
Source: Nucleic Acids Res. 2026 Aug 26;54(16):gkag852. doi: 10.1093/nar/gkag852 (PMC13507859; doi:10.1093/nar/gkag852)
Supplement: Supplementary file 1 [file gkag852_supplemental_file.pdf]

Supplementary materials for:

Genetic studies with a uracil DNA glycosylase biosensor support a role for mitochondrial UNG1 in nuclear uracil repair

Yu-Hsiu T. Lin[1], Analisa M. Lott[1], Xingyu Liu[1], Layla Abdulbaki[1], Yanjun Chen[1], Michael A. Carpenter[1,2], Reuben S. Harris[1,2,*]

[1] Department of Biochemistry and Structural Biology, University of Texas Health San Antonio, San Antonio, TX 78229, USA

[2] Howard Hughes Medical Institute, University of Texas Health San Antonio, San Antonio, TX 78229, USA

[*] Correspondence: rsh@uthscsa.edu

Key words: C-to-U deamination; cytosine deamination; uracil base excision repair (UBER); uracil-DNA *N*-glycosylase (UNG); uracil glycosylase inhibitor (Ugi)

Supplementary Tables S1-S2

Supplementary Figures S1-S6

Supplementary Table S1. Oligonucleotide sequences.

Oligo name	Purpose	Sequence 5' to 3'
hUNG2-insert-cloning-F	Forward PCR primer for amplifying hUNG2 followed by restriction cloning into pcDNA3.1	CTTAAGCTTGCCACCATGATCGGCCAGAAG
hUNG2-insert-cloning-R	Reverse PCR primer for amplifying hUNG2 followed by restriction cloning into pcDNA3.1	GCAGAATTCCCTACAGCTCCTTCCAG
remove-Ugi-SDM-F	Forward SDM primer for removing Ugi's from A3Bctd-BE4max	GAGGTGACTCTGGCGGCTCAAAA
remove-Ugi-SDM-R	Reverse SDM primer for removing Ugi's from A3Bctd-BE4max	CCGCCAGAGTCACCTCCCAGCTG
Ugi-insert-F	Forward PCR primer for amplifying Ugi insert for InFusion cloning	GGATCCGCCACCATGACTAATCTGAGCGACATC
Ugi-insert-R	Reverse PCR primer for amplifying Ugi insert for InFusion cloning	TCTGCAGAATTCTTACAGCATCTTGATCTTATTC
Ugi-backbone-F	Forward PCR primer for amplifying pcDNA3.1 backbone for InFusion cloning	CATGGTGGCGGATCCGAGCTCGGT
Ugi-backbone-R	Reverse PCR primer for amplifying pcDNA3.1 backbone for InFusion cloning	TAAGAATTCTGCAGATATCCAGC
Ugi-FLAG-SDM-F	Forward SDM primer to insert FLAG sequence into C-terminus of Ugi	GACTACAAGGACGACGATGACAAGTA-AGAATTCTGCAGATATCCAGCACAGTGG
Ugi-FLAG-SDM-R	Reverse SDM primer to insert FLAG sequence into C-terminus of Ugi	CTTGTCATCGTCGTCCTTGTAGTCCAG-CATCTTGATCTTATTCTCGCCGTTAG
hUNG2-PCR-F	Forward primer for generating amplicon from gDNA to confirm human *UNG2* KO	CACTGGGCCTTCCACCG
hUNG2-PCR-R	Reverse primer for generating amplicon from gDNA to confirm human *UNG2* KO	CTCTGATTGGACAGCGCACC
hUNG1-PCR-F	Forward primer for generating amplicon from gDNA to confirm human *UNG1* and *UNG1/2* KO	GGTGCGCTGTCCAATCAGAG
hUNG1-PCR-R	Reverse primer for generating amplicon from gDNA to confirm human *UNG1* and *UNG1/2* KO	TGGCCGGCTACACTAACAAG
remove-Ugi-sequencing	Sequencing primer to confirm Ugi removal from A3Bctd-BE4max	GCGATGCAATTTCCTCATTT
pcDNA3.1-sequencing	Sequencing primer to confirm inserts in pcDNA3.1 vectors	CGCAAATGGGCGGTAGGCGTG
pJET12F-sequencing	Sequencing primer to confirm inserts in pJET vectors	CGACTCACTATAGGGAGAGCGGC

Supplementary Table S2. *UNG* genotypes of knockout clones*.

Cells	Coding mRNA	Protein	Mutant allele 1	Mutant allele 2	Mutant allele 3
293T *UNG1* KO c7	NM_003362.4 (*UNG1*)	NP_003353.1 (*UNG1*)	c.25_38del/p.(Trp9GlufsTer120) [2,739 reads]	c.30del/p.(Leu11TrpfsTer16) [2,601 reads]	
293T *UNG1* KO c30	NM_003362.4 (*UNG1*)	NP_003353.1 (*UNG1*)	c.25_38del/p.(Trp9GlufsTer120) [5,331 reads]		
293T *UNG2* KO c11	NM_080911.3 (*UNG2*)	NP_550433.1 (*UNG2*)	c.3_5del/p.0 [1,664 reads]	c.[3_5del;32del]/p.0 [344 reads]	c.2_132+218delinsGCTG/p.0 [Sanger]
293T *UNG2* KO c12	NM_080911.3 (*UNG2*)	NP_550433.1 (*UNG2*)	c.2_7del/p.0 [2,496 reads]	c.-9_1del/p.0 [2,392 reads]	c.1_2ins[468bps]/p.0 [Sanger]
293T *UNG1/2* KO c13	NM_080911.3 (*UNG2*)	NP_550433.1 (*UNG2*)	c.262_272delinsA/p.(Arg88ThrfsTer24) [2,696 reads]	c.264_274del/p.(Asn89GlyfsTer50) [2,545 reads]	
293T *UNG1/2* KO c52	NM_080911.3 (*UNG2*)	NP_550433.1 (*UNG2*)	c.237_275delinsGGGGG/p.(Ala80GlyfsTer24) [5,291 reads]	c.273dup/ p.(Val92ArgfsTer51) [Sanger]	c.237_274del/ p.(Ala80GlyfsTer50) [Sanger]
U2OS *UNG1* KO c6	NM_003362.4 (*UNG1*)	NP_003353.1 (*UNG1*)	c.25_38del/p.(Trp9GlufsTer120) [3,529 reads]		
U2OS *UNG1* KO c7	NM_003362.4 (*UNG1*)	NP_003353.1 (*UNG1*)	c.27_30delinsTTCCACAAATGGGCGTCTTCTGCC/p.(Trp9CysfsTer25) [4,670 reads]		
U2OS *UNG2* KO c2	NM_080911.3 (*UNG2*)	NP_550433.1 (*UNG2*)	c.1_88del/p.0 [2,969 reads]	c.-1_12del/p.0 [2169 reads]	
U2OS *UNG2* KO c7	NM_080911.3 (*UNG2*)	NP_550433.1 (*UNG2*)	c.-1_12del/p.0 [4,348 reads]		
U2OS *UNG1/2* KO c3	NM_080911.3 (*UNG2*)	NP_550433.1 (*UNG2*)	c.272_273insT/p.(Val92ArgfsTer51) [4,713 reads]		
U2OS *UNG1/2* KO c8	NM_080911.3 (*UNG2*)	NP_550433.1 (*UNG2*)	c.267_295del/p.(Asn89LysfsTer44) [2,098 reads]	c.241_274del/p.(Ala81TrpfsTer23) [2,039 reads]	

* 293T and U2OS have abnormal karyotypes with 3 and 2 copies of the *UNG* locus, respectively. Based on our sequence analyses, clones with fewer alleles are likely due to identical CRISPR resolutions, loss-of-heterozygosity during CRISPR-mediated genome engineering, and/or to deletions that extend beyond the interval analyzed by PCR and sequencing (*i.e.*, large null alleles).

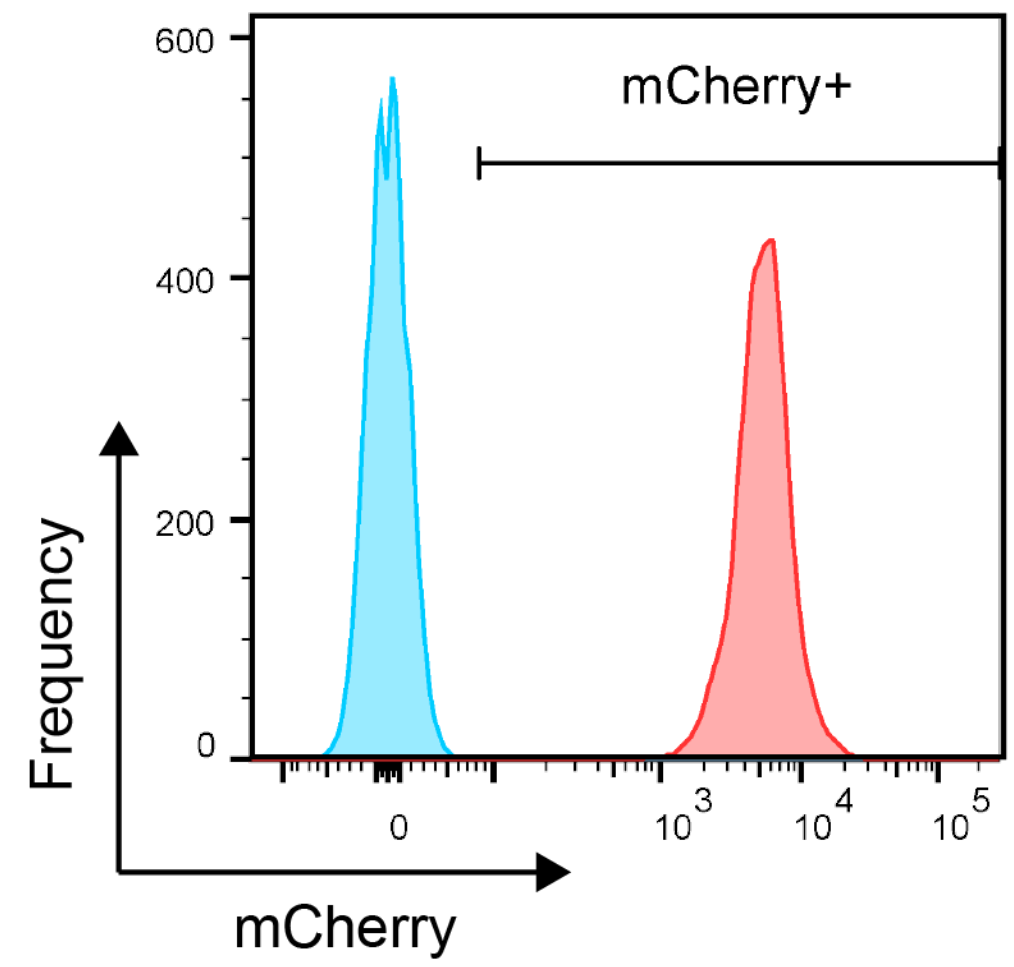

Supplementary Figure S1. Flow cytometry analysis of 293T-cAMBER c15.

Flow analysis of parental 293T cells (blue) and 293T-cAMBER c15 cells (red), which has the AMBER construct integrated stably into chromosomal DNA. The precise insertion site has yet to be identified but is likely single copy based on uniformly high mCherry expression levels.

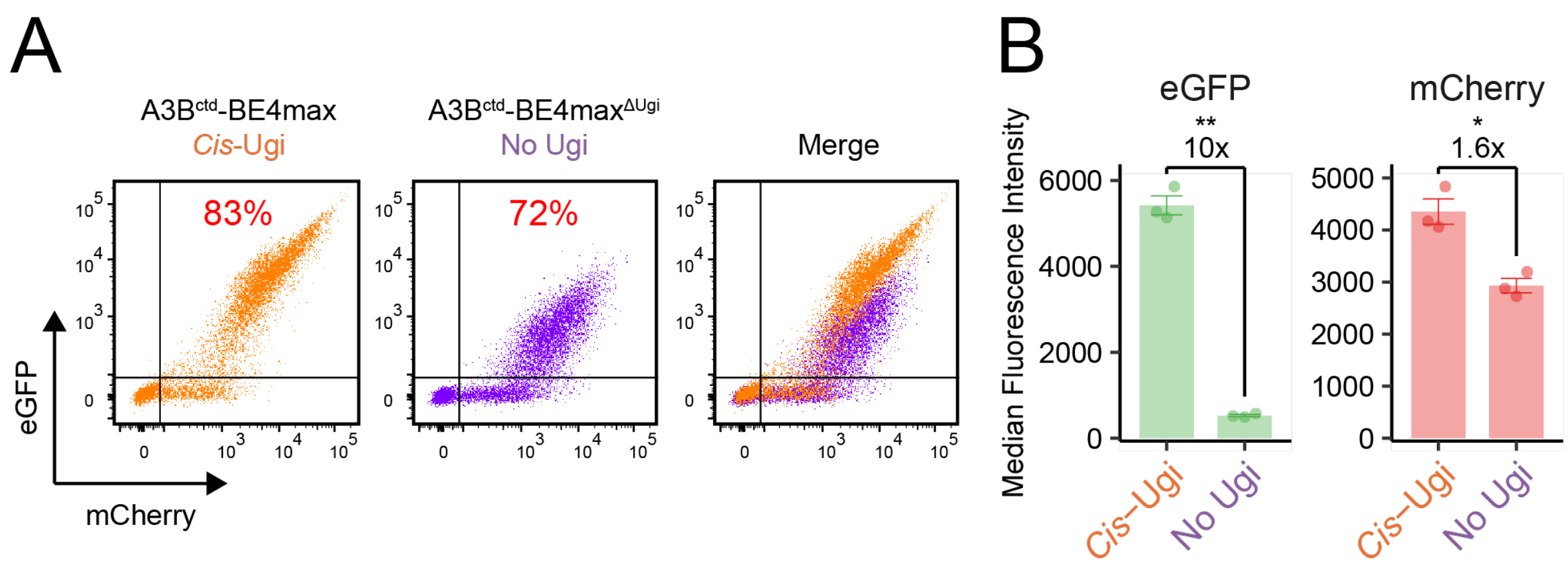

Supplementary Figure S2. Flow cytometric confirmation of changes in fluorescence upon Ugi removal.

A-B) Flow analysis and quantification of the singlet population harvested 48 hours post-transfection showing reduced eGFP and mCherry levels when Ugi is removed. The percentages in red indicate the ratio double-positive to mCherry-positive cell populations (*n*=3, mean±SEM).

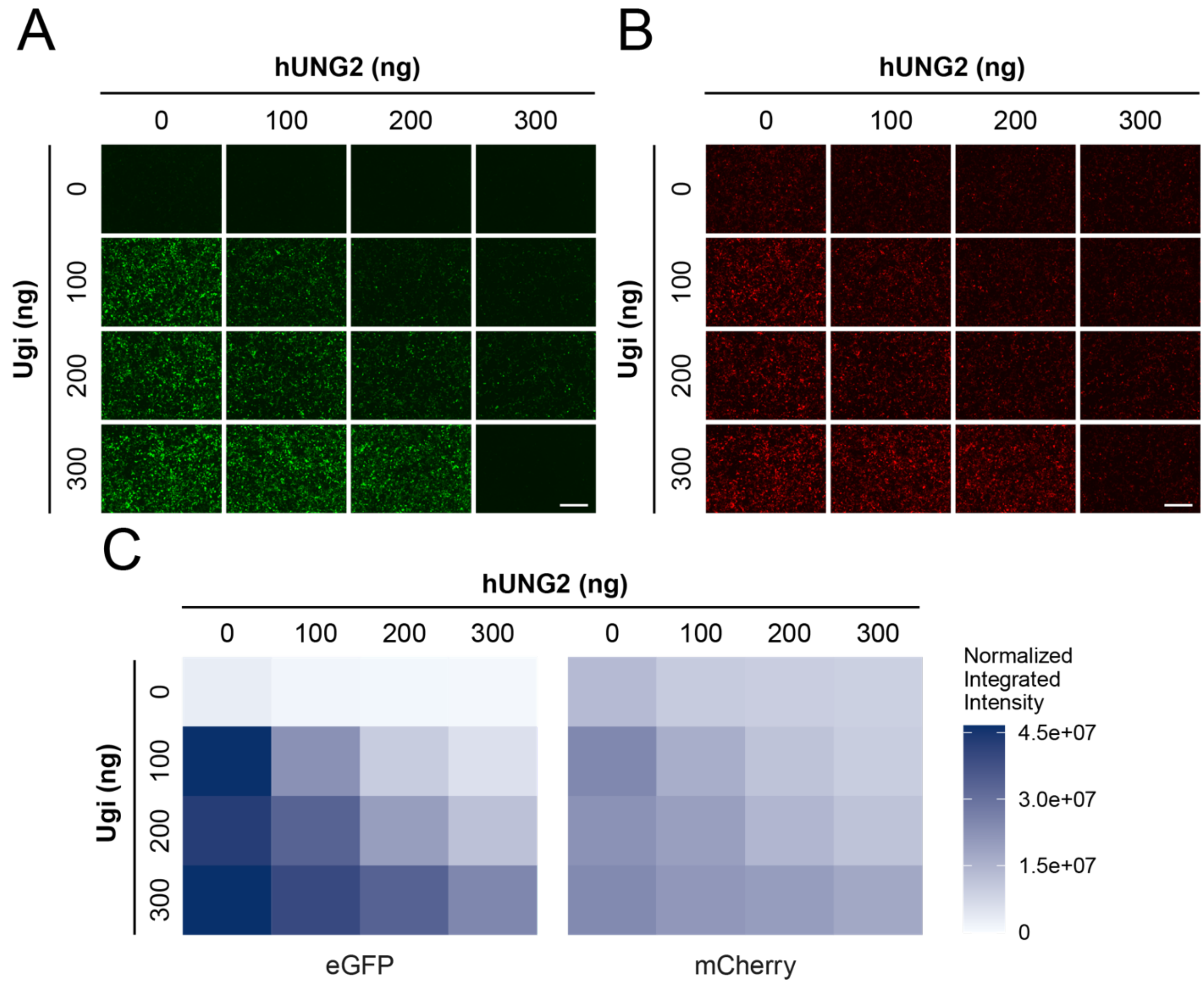

Supplementary Figure S3. U-report biosensor is responsive to varying levels of UNG activity.

A-B) Fluorescence microscopy of eGFP (left) and mCherry (right) signals 48 hours post-transfection of 293T cells with Ugi and human UNG2 (hUNG2) in the episomal system (scale=400µm).

C) Quantification of the fluorescence images at 48 hours post-transfection.

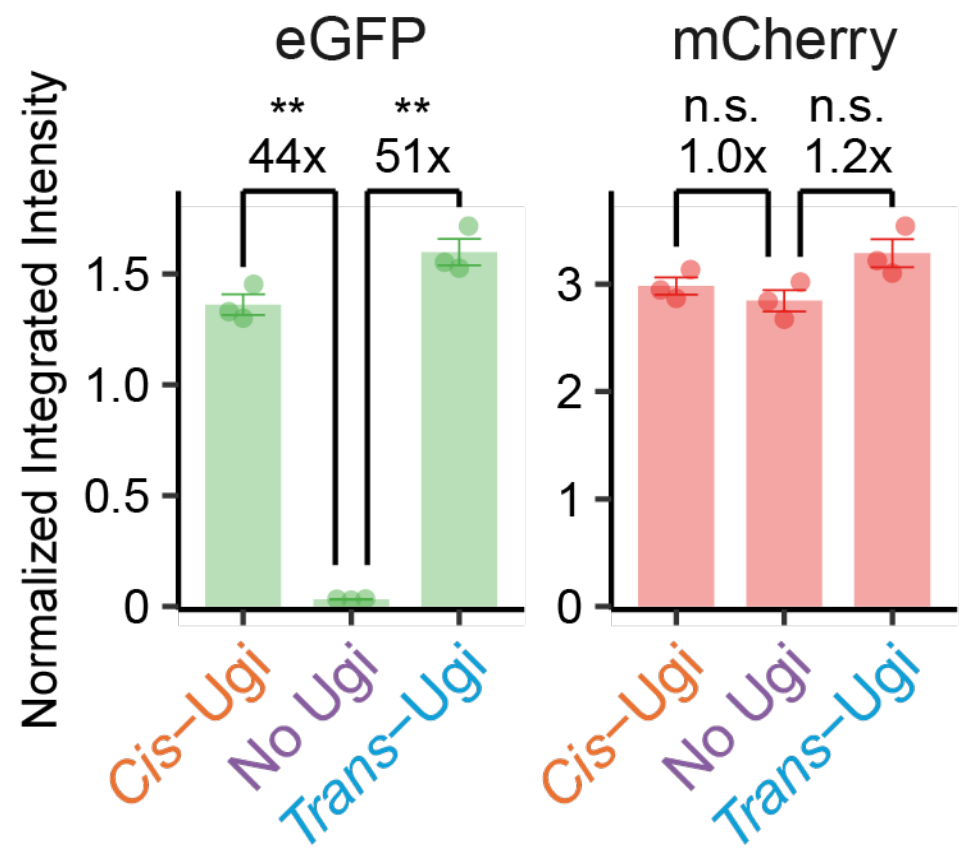

Supplementary Figure S4. Quantification of fluorescence imaging of the chromosomal U-report system.

Comparison of the normalized integrated intensities of eGFP and mCherry across the three conditions in the 293T-chromosomal AMBER c15 cells at 48 hours post-transfection (*n*=3, mean±SEM).

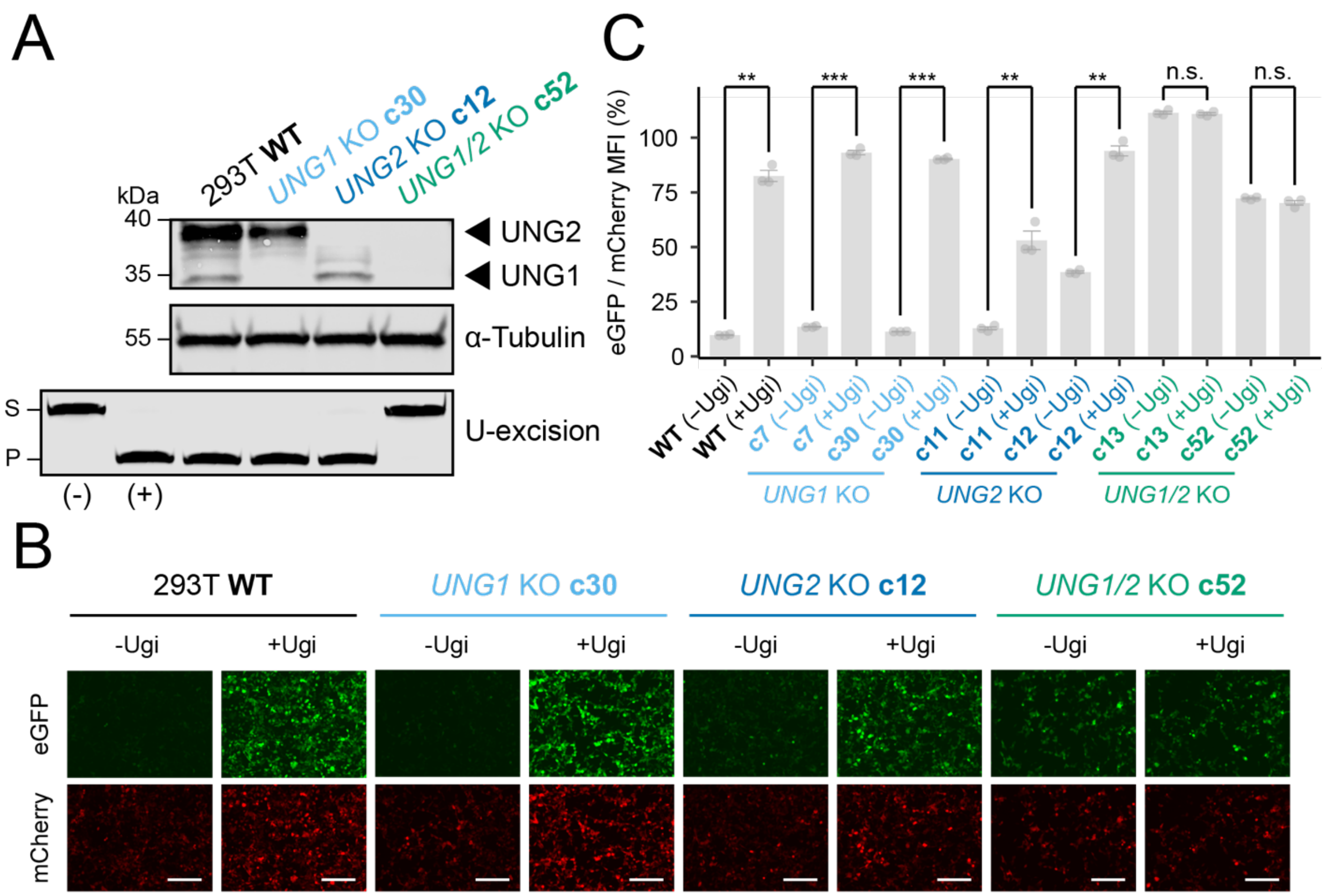

Supplementary Figure S5. U-report biosensor in 293T *UNG* KO clones.

A) Immunoblot and *in vitro* uracil excision assay confirming *UNG* inactivation in 293T cells [S, uracil-containing 43-mer ssDNA substrate; P, cleaved 30-mer ssDNA product; (-) = HED buffer only (negative control); (+) = 5 Units of *E. coli* uracil-DNA glycosylase (positive control for uracil excision)].

B) Fluorescence microscopy of additional *UNG* KO clones in 293T cells at 48 hours post-transfection (scale=200µm).

C) Ratio of eGFP to mCherry median fluorescence intensities at 48 hours post-transfection shows full UNG inhibition only in *UNG1/2* double-KO clones (*n*=3, mean±SEM). Quantification and statistical comparisons are only relevant, as indicated, for individual clones (minus/plus Ugi) due to the variability of transfection efficiencies between independent clones.

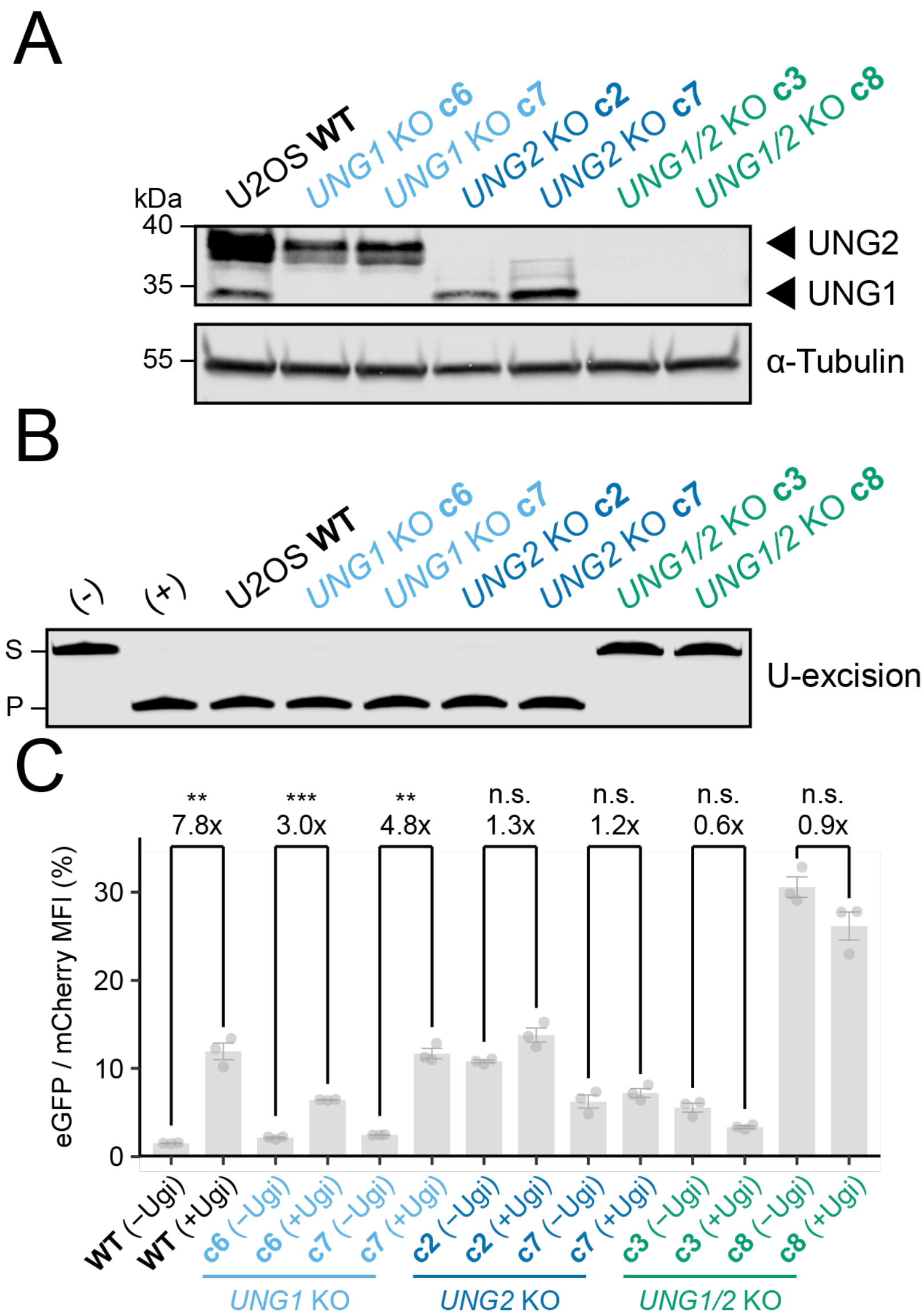

Supplementary Figure S6. U-report biosensor in U2OS *UNG* KO clones.

A) Immunoblot validation of *UNG* isoform disruption in U2OS cells.

B) Uracil excision activity in whole cell extracts from the indicated clones [S, uracil-containing 43-mer ssDNA substrate; P, cleaved 30-mer ssDNA product; (-) = HED buffer only (negative control); (+) = 5 Units of *E. coli* uracil-DNA glycosylase (positive control for uracil excision)].

C) Ratio of eGFP to mCherry median fluorescence intensities at 48 hours post-transfection using the U-report system. Fold changes in the eGFP-to-mCherry MFI between the presence and absence of Ugi are indicated (*n*=3, mean±SEM). Quantification and statistical comparisons are only relevant, as indicated, for individual clones (minus/plus Ugi) due to the variability of transfection efficiencies between independent clones.
